# Using short video-based educational intervention to enhance initial acceptance to continuous positive pressure therapy in Thai obstructive sleep apnea patients in a tertiary care: a randomized controlled trial

**DOI:** 10.1007/s11325-025-03419-8

**Published:** 2025-07-16

**Authors:** Jindapa Srikajon, Chawanont Pimolsri, Chatchawan Rattanabannakit, Wattanachai Chotinaiwattarakul

**Affiliations:** 1 Siriraj Sleep Center, Siriraj Hospital, Mahidol University, 2 Wanglang Road, Bangkoknoi, Bangkok, 10700 Thailand; 2 Division of Neurology, Department of Medicine, Faculty of Medicine, Siriraj Hospital, Mahidol University, Bangkok, 10700 Thailand

**Keywords:** Obstructive sleep apnea syndrome, Continuous positive airway pressure, Adherence

## Abstract

**Purpose:**

Obstructive sleep apnea (OSA) is a prevalent condition associated with decreased quality of life and increased risk of cardiovascular complications. Continuous positive airway pressure (CPAP) therapy is the gold standard treatment, particularly in moderate to severe OSA. Its effectiveness depends on patient adherence, with compliance rates ranging from 46 to 83%. Various strategies, including education, cognitive behavioral therapy, and supportive care, aim to improve CPAP adherence. This study evaluated the effectiveness of a low-cost, time-efficient video-based educational intervention to enhance CPAP compliance.

**Methods:**

Patients diagnosed with moderate to severe OSA or mild OSA who fulfilled the indication for CPAP therapy in Siriraj Hospital between December 1st, 2022, and May 31st, 2023, age ≥ 18 years, were randomized into the three-minute video intervention group, based on Albert Bandura’s social cognitive theory, and usual care group using a simple random sampling method. Demographics, Epworth Sleepiness Scale scores (ESS), polysomnographic data, and CPAP usage were recorded.

**Results:**

Of the 132 subjects in this study, the majority were male, with an average age of 50 and comparable body mass index (BMI) and baseline ESS in both groups. We found a significantly higher percentage meeting the Centers for Medicare & Medicaid Services (CMS) criteria for adherence in the video education group (68.2%, vs. 50%; *p* = 0.034) compared to the usual care group. Moreover, in our post-hoc analysis, higher education years and lower BMI were associated with better CPAP adherence.

**Conclusion:**

Our data demonstrated a promising result of using a short-formed video education intervention to enhance CPAP usage in OSA patients. Longer-term studies with a larger population would provide more reliable outcomes. This trial was retrospectively registered with Thaiclinicaltrials.org. The trial registration number (TRN) was TCTR20240619006, and the registration date is 18 June 2024.

## Introduction

The prevalence of obstructive sleep apnea (OSA) is increasing globally. Continuous positive airway pressure (CPAP) therapy is the most widely used and effective treatment for moderate and severe OSA. CPAP has been shown to enhance sleep quality and reduce the risk of cardiovascular events such as stroke and myocardial infarction [1].

One of the primary challenges in CPAP therapy is ensuring patient compliance. Which is defined as usage for at least 4 h per night on at least 70% of nights [2]. Greater nightly usage is associated with improved functional outcomes, including reduced daytime sleepiness and enhanced quality of life [3, 4]. Nevertheless, studies have reported subjective adherence rates of 65-90%, while objective measures of CPAP adherence range from 40 to 83% [5].

There are several ways to enhance adherence to CPAP in patients with OSA, such as education, behavioral therapy, or supportive care. Among these, the educational strategy is one of the most commonly utilized for CPAP-naïve patients [5, 6]. The American Academy of Sleep Medicine strongly recommends implementing educational interventions before starting PAP therapy in adults with OSA [7].

Despite the mixed results of the previous studies, we still decided to evaluate the impact of educational videos on CPAP usage in Thai patients. To the best of our knowledge, this is the first study about the effectiveness of video education in CPAP adherence improvement in the Thai population. In our opinion, educational video is a time-saving method to enhance CPAP usage in OSA patients. Not only is this intervention low-cost and less time-consuming but it can also be adapted and used in other healthcare centers in Thailand. Thus, we developed a short-formed educational video in the Thai language and compared the percentage of good CPAP users between the group with the educational video and the group that did not watch it.

## Materials and methods

### Study design

This single-center randomized controlled trial was conducted at the Siriraj Sleep Center, Faculty of Medicine, Siriraj Hospital, Bangkok, Thailand. Ethical approval was obtained from the Human Research Ethics Committee of Siriraj Hospital, Mahidol University (approval number 042/2566). The primary objective was to evaluate the impact of video-based education on initial CPAP adherence rates among OSA patients. Secondary objectives included identifying predictors of successful CPAP adherence.

### Study participants

We recruited adult participants (aged ≥ 18 years) diagnosed with moderate to severe OSA or mild OSA requiring CPAP therapy, as determined by standard polysomnography (PSG), between December 1, 2022, and May 31, 2023, at Siriraj Sleep Center, Siriraj Hospital, Bangkok, Thailand. OSA was diagnosed in accordance with the American Academy of Sleep Medicine Task Force guidelines [8]: mild (apnea-hypopnea index [AHI] 5–15 events/h), moderate (AHI 15–30 events/h), and severe (AHI ≥ 30 events/h).

Exclusion criteria included prior CPAP usage (non-naïve), a diagnosis of central sleep apnea, contraindications to CPAP therapy due to comorbidities, or refusal to use CPAP.

The sample size was estimated at 132 participants based on the average proportion of good CPAP users with standard care from our center’s database to be around 50%. To detect an improved difference of proportion of 25%, calculated by referring to the percentage of effective CPAP users in a prior study conducted in Turkey after an educational video intervention [9], approximately 66 patients would be required per group, based on a two-group comparison with 80% power and two-tailed significance of 0.05.

### Polysomnographic study

All participants underwent in-laboratory PSG at the Sleep Center of Siriraj Hospital using the SOMNO HD EEG32 System (SOMNOmedics AG, Medical equipment manufacturer in Randersacker, Germany). PSG studies were performed and analyzed according to the American Academy of Sleep Medicine scoring manual [10]. The oxygen desaturation index was defined as the number of oxygen desaturations of > 3% per hour of sleep. OSA severity was classified as already mentioned.

### Procedure

1. Participants provided informed consent during their post-polysomnography (PSG) appointment.
2. Baseline demographic data, PSG results, and questionnaire scores, including the Epworth Sleepiness Scale (ESS), Fatigue Severity Scale (FSS), and Pittsburgh Sleep Quality Index (PSQI), were collected.
3. Participants were randomly assigned to either the video education intervention group or the usual care group using a permuted block randomization method with two different block sizes (4 and 6). We prepared blocks of 4 and 6 by using envelopes with the group labels inside, which were shuffled and allocated during each visit. The randomization and allocation process was conducted by a research assistant who was concealed from the principal investigator.
4. Participants in the video education group watched a three-minute video covering OSA, CPAP therapy, and its benefits. The video included testimonials from current CPAP users to enhance motivation. Participants in this group also received routine care.
5. The usual care group received standard verbal counseling from sleep medicine physicians and technicians, who provided information on OSA, CPAP use, mask fitting, and troubleshooting.
6. All participants were scheduled for a follow-up visit three weeks after initiating CPAP therapy.
7. CPAP usage data and updated questionnaire scores were collected during the follow-up visit.

### Educational video intervention

**Fig. 1:**
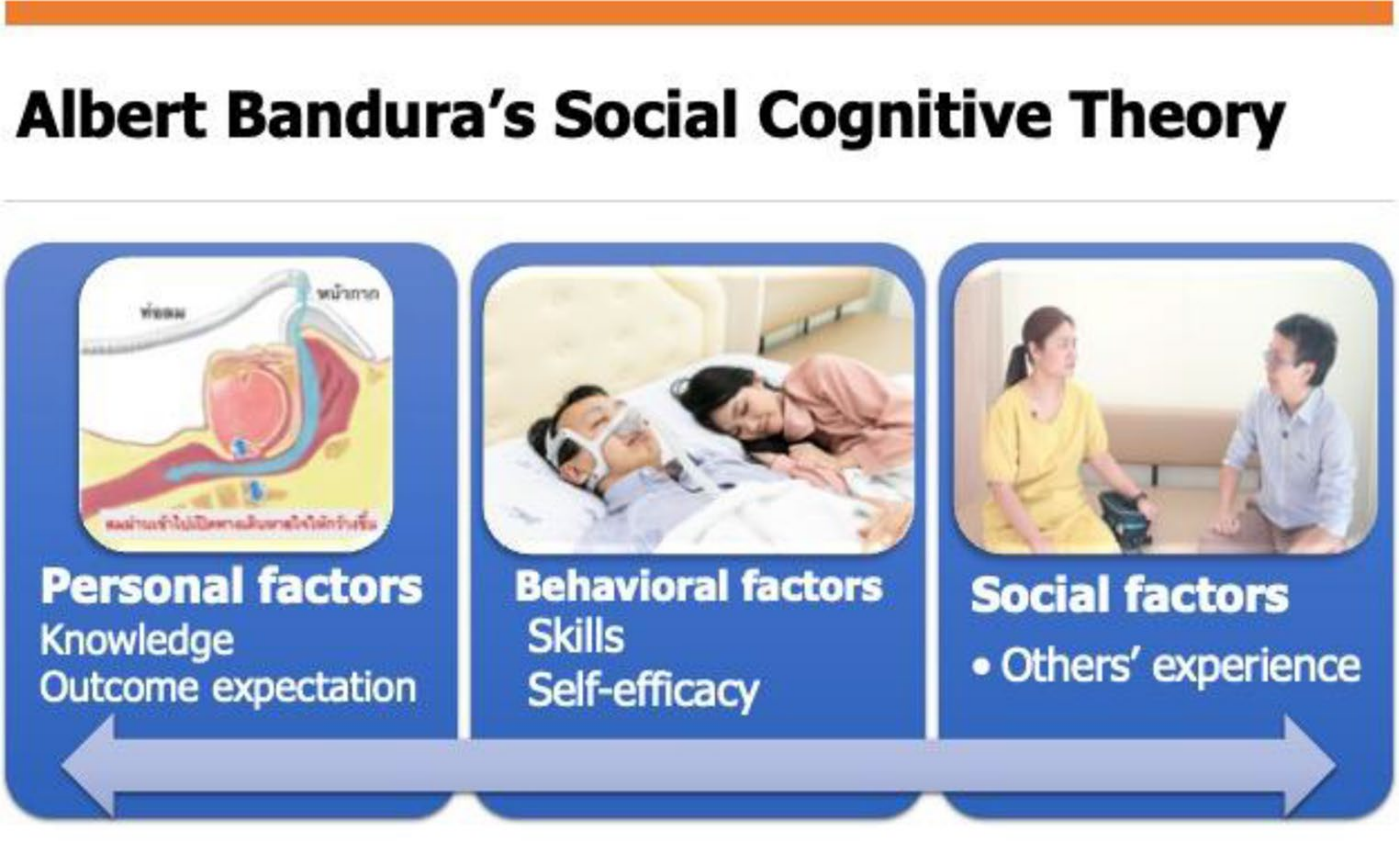
Components of the educational video based on Albert Bandura’s Social Cognitive Theory

Research assistants guided participants in the intervention group to a private, quiet room where the educational video was played on a notebook computer. The three-minute video, based on Albert Bandura’s social cognitive theory, which comprises self-efficacy, outcome expectations, and social support, was developed with input from sleep medicine specialists and behavioral neurologists [11]. The multidisciplinary team aims to improve self-efficacy and facilitate them to change their perception by showing them apparent outcomes (Fig. 1).

The 3-minute video was divided into four segments. The first two parts provided basic knowledge about OSA, including its pathophysiology and the benefits of CPAP therapy. We elaborated on the long-term health benefits, such as reduced sleepiness, improved memory and mood, and the prevention of cardiovascular risks. A demonstration showing how to wear a CPAP mask was also included to explain the simple process of CPAP use. The next part featured real-life testimonials from two CPAP users of different genders, sharing their personal experiences and how using CPAP is worthwhile. This section focuses on motivating participants while addressing common barriers to adherence. The last part briefly introduces CPAP’s adverse effects and troubleshooting. We intentionally made this segment the shortest and ended the video by reassuring viewers about the outcomes and how patients will eventually adjust to the machine. The Thai language was used in both the narrative voice and subtitles, avoiding any non-technical language. The contents and goals of each section are listed in Table 1. Participants in the video group also received standard care, as described below.

**Table 1:** Video content and goals

Video section	1.General knowledge about OSA	2. What is CPAP and its benefits	3. Scenario	4.CPAP troubleshooting
Main content	Introduction to the pathophysiology and symptoms of OSA and how it affect overall health and quality of life	The function of CPAP and how it helps alleviate the symptoms and consequences of OSA and demonstration of how to put on a CPAP mask	A real conversation between OSA patients who successfully use CPAP	Clarify some CPAP adverse events i.e. mask leakage and dry mouth and reassure that they will eventually get used to the machine
Goals	Improve basic knowledge	Enhance outcome expectation	Increase self-efficacy and outcome expectation	Increase self-efficacy and social support
Duration	1:20 min	1:00 min	40 s	30 s

### Standard care

Following their PSG study, all participants were seen by a sleep physician and provided with standard verbal counseling on OSA and CPAP therapy. Participants received a prescription for a CPAP machine, including specific device settings and an appropriate mask type.

Trained sleep technicians demonstrated CPAP operation, proper mask fitting, and troubleshooting. Participants were also educated on managing common challenges associated with CPAP use. Three weeks after initiating therapy, a follow-up appointment at the sleep clinic was scheduled.

### Outcome analysis and patient follow-up

The primary outcome was the percentage of participants meeting the criteria for good adherence [2] at three weeks, defined as CPAP usage for ≥ 4 h per night on at least 70% of nights. This outcome was used to assess initial CPAP acceptance. CPAP usage data from the past three weeks were retrieved from the device’s memory card during the trial period.

Secondary outcomes included the average nightly CPAP usage (hours per night) over the three-week period and the percentage of days with ≥ 4 h of CPAP use. These metrics were compared between the video education and usual care groups.

Predictors of good CPAP adherence were also evaluated by analyzing changes from baseline to three weeks in the following measures: Epworth Sleepiness Scale (ESS), Pittsburgh Sleep Quality Index (PSQI), and apnea-hypopnea index (AHI).

### Statistical analysis

All statistical analyses were performed using Statistical Package for Social Sciences, version 20.0 for the Windows system (SPSS Inc., Mahidol University, Thailand). Continuous variables were summarized as mean ± standard deviation (SD), while categorical variables were expressed as frequencies and percentages.

Group comparisons were conducted using the Student’s t-test or Mann-Whitney U test for continuous variables, depending on data distribution. Categorical variables were analyzed using the chi-square test.

Univariate logistic regression was employed to identify demographic, gender, socioeconomic status, and polysomnographic variables potentially associated with initial CPAP acceptance. Variables with a p-value < 0.1 in univariate analysis were subsequently entered into a multivariate logistic regression model using the enter method. Adjusted odds ratios (ORs) with 95% confidence intervals (CIs) were reported.

Statistical significance was set at *p* < 0.05.

## Results

**Fig. 2:**
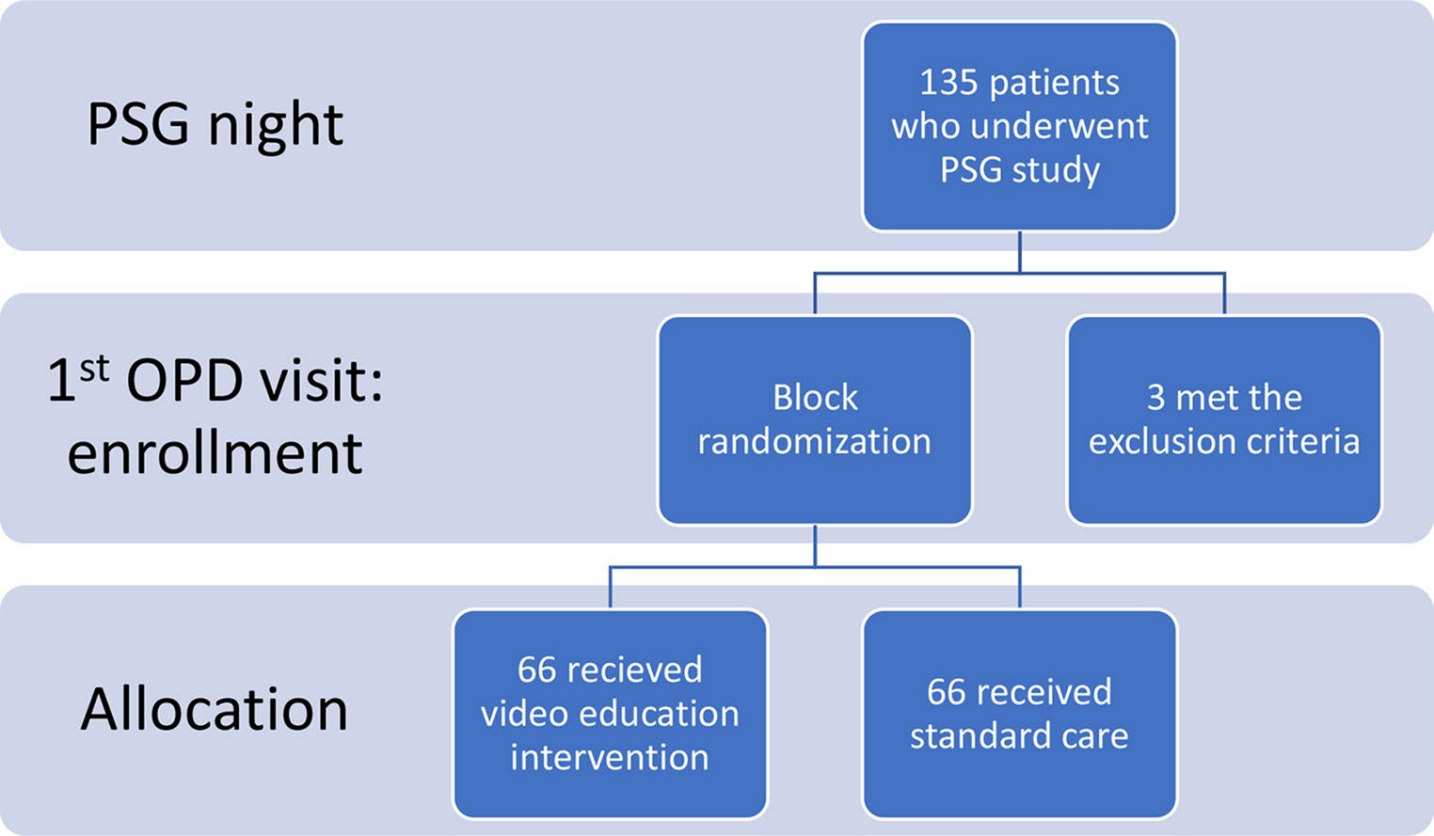
Flow chart

This single-center randomized controlled trial included 132 adults diagnosed with OSA and indicated for CPAP therapy, recruited from Siriraj Sleep Center between December 1, 2022, and May 31, 2023. Of the 135 patients who met eligibility criteria and consented to participate, 3 were excluded due to refusal to use CPAP therapy. The remaining 132 participants were randomized equally into the video education group (*n* = 66) and the usual care group (*n* = 66) (Fig. 2).

### Baseline characteristics

Baseline demographic and clinical characteristics were similar between the video education and usual care groups. The majority of participants were male, with an average age of 50 years. The prevalence of hypertension and diabetes mellitus was comparable between groups. No significant differences were observed in baseline Epworth Sleepiness Scale (ESS) scores or Pittsburgh Sleep Quality Index (PSQI) scores. The intervention group had a slightly higher proportion of patients under the Civil Servant Medical Benefit Scheme (CSMBS) compared to the control group, without statistical significance. Detailed demographic data are presented in Table 2.

**Table 2:** Baseline characteristics of participants

Factors (Median(min, max))	Video education (*n* = 66)	Usual care (*n* = 66)	*P*-value
Age (years)	51.7 ± 13.66	50.2 ± 16.26	0.550
Male, n (%)	41 (62.1)	40 (60.6)	0.858
BMI (kg/m^2^)	28.7 (18.7, 59)	29.3 (17.7, 67.7)	0.647
Civil Servant Medical Benefit Scheme (CSMBS), n(%)	36 (54.5)	30 (45.5)	0.296
Diabetes mellitus (%)	10 (15.2)	16 (24.2)	0.189
Hypertension (%)	24 (36.4)	27 (40.9)	0.347
Diabetes mellitus (%)	13 (19.7)	20 (30.3)	0.159
Stroke (%)	3 (4.5)	5 (7.6)	0.718
Marital status (%)			
- Single	24 (36.3)	27 (40.9)	0.746
- Married	40 (60.6)	36 (54.5)	
- Divorced	2 (3)	3 (4.5)	
Have bed partner (%)	37 (56)	35 (53)	0.727
Education (years)	16 (4, 20)	16 (4, 18)	0.498
Visit with family (%)	15 (22.7)	17 (25.8)	0.685
Epworth sleepiness score (ESS)	8 (0, 23)	9 (2, 18)	0.680
Pittsburgh Sleep Quality Index (PSQI)	8.08 ± 3.47	7.83 ± 3.23	0.672
Fatigue Severity Scale (FSS)	29.89 ± 13.9	31.4 ± 13.3	0.534

### Polysomnography findings and CPAP usage data

Baseline polysomnography (PSG) data are shown in Table 3. Overall, the study mostly included moderate to severe OSA. The median AHI was 49.4 (5.1-166.2) in the intervention group and 42.2 (7.3, 146.3) in the usual care group. 3% Oxygen Desaturation Index scores and sleep efficiency were similar in the two groups. The PAP settings, type of mask, and leakage on the home PAP devices were not significantly different. The rest PAP usage variables are summarized in Table 4.

**Table 3:** Polysomnographic data

Factors	Video education (*n* = 66)	Usual care (*n* = 66)	*P*-value
PSG type (%)			
- Full night	33 (50)	40 (54.8)	0.220
- Split night	33 (50)	26 (44.1)	
AHI (events/h)	49.4 (5.1, 166.2)	42.2 (7.3, 146.3)	0.265
LSpO_2_	82.5 (38, 94)	84 (47, 96)	0.347
3%ODI	29.4 (1.2, 131.9)	24.8 (0, 145.1)	0.196
SE (%)	81.2 (24.3, 97.4)	75.7 (35.6, 98.6)	0.346
Arousal index	35.6 (5.4, 100.9)	32.8 (3.9, 120.9)	0.323

**Table 4:** PAP usage data

Factors	Video education (*n* = 66)	Usual care (*n* = 66)	*P*-value
Mask type (%)			
- Nasal	61 (92.4)	66 (100)	0.058
- Full face	5 (7.6)	0	
PAP brand (%)			
- Respironics	18 (27.3)	23 (34.8)	0.409
- Apex	47 (71.2)	43 (65.2)	
- Phillips	1 (1.5)	0	
PAP setting type			
- CPAP setting (%)	16 (24.2)	13 (19.7)	0.528
- APAP setting (%)	50 (75.8)	53 (80.3)	
CPAP pressure (cmH2O)	11 (4, 16)	11 (6, 20)	0.853
APAP min	5 (4, 8)	15 (10, 20)	0.648
APAP max	5(4, 8)	15 (10, 20)	0.802
APAP P95 pressure	7 (4.9, 19.6)	7.4 (4.9, 13.7)	0.65
Residual AHI	1.7 (0.1, 10.8)	1.4 (0, 29.9)	0.144
Leakage (LPM)	19.4 (0, 64.6)	21.3 (0, 87.6)	0.359
EPR	10 (15.2)	4 (6.1)	0.090

Data presented as median (interquartile range) or number (percentage)

### Initial CPAP acceptance

**Fig. 3:**
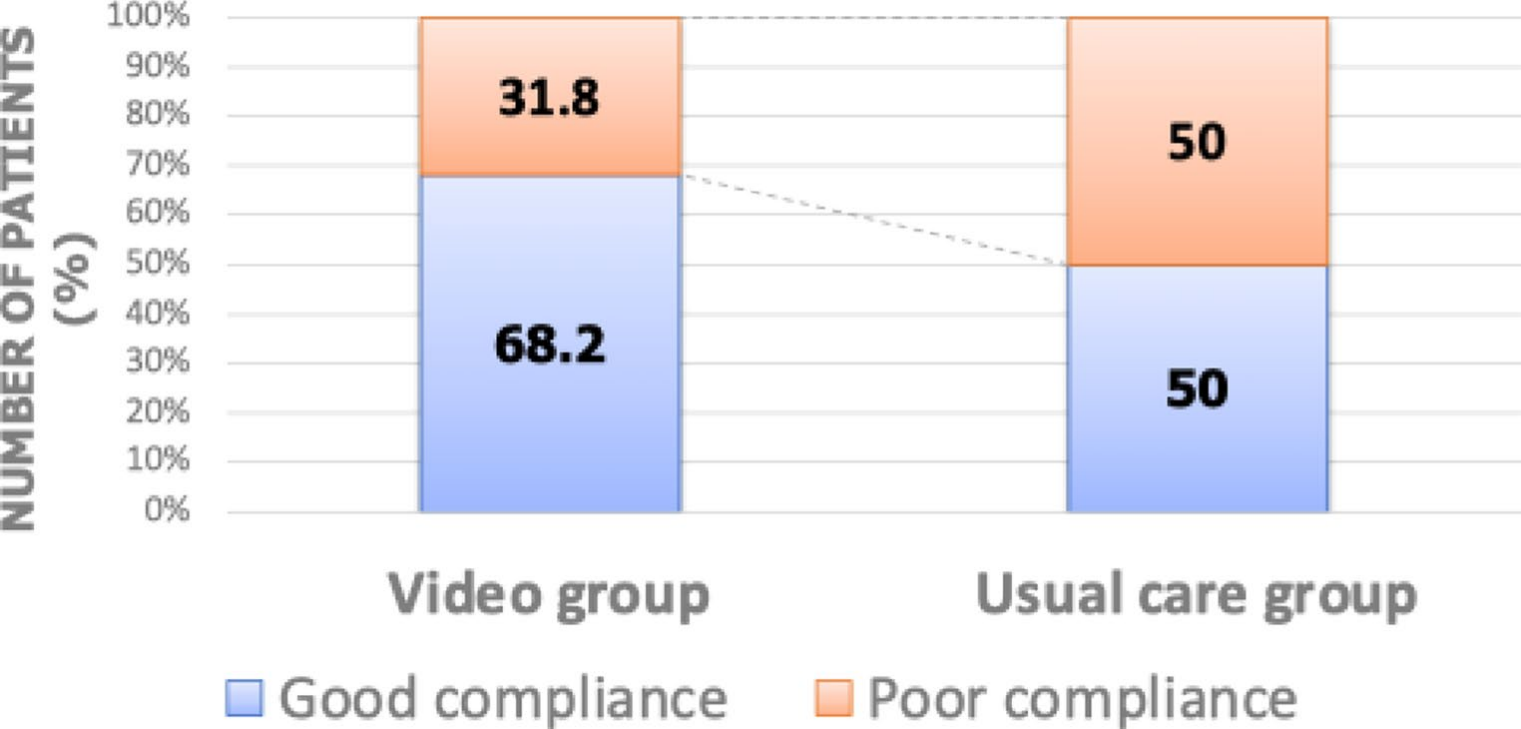
Percentage of good CPAP compliance

The primary outcome, adherence to CPAP therapy as defined by CMS criteria, was significantly higher in the video education group (68.2%) compared to the usual care group (50%; *p* = 0.034) at the three-week follow-up (Table 5; Fig. 3).

**Table 5:** Primary and secondary outcome

Outcomes	Video education (*n* = 66)	Usual care (*n* = 66)	*P*-value
Percent meeting criteria for good adherence, n (%)	45 (68.2)	33 (50)	0.034
Mean daily CPAP use, hour/night	6 (0.3, 9.3)	4.5 (0.2, 9)	0.012
Percent days above 4-hour use	87.4 (0, 100)	68.9 (0, 100)	0.005

**Fig. 4:**
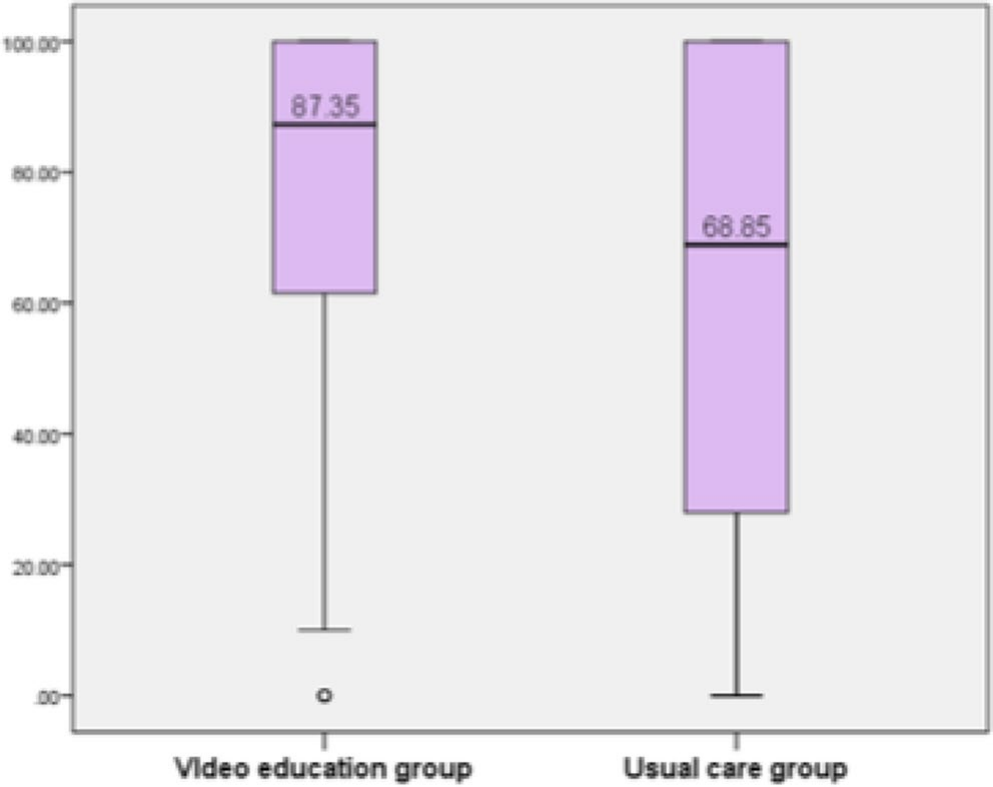
Days above 4-hour CPAP use (%)

For secondary outcomes, the video education group demonstrated significantly greater average CPAP usage (mean 6 h/night vs. 4.5 h/night, *p* = 0.012) and a higher percentage of days with ≥ 4 h of CPAP use (87.4% vs. 68.9%; *p* = 0.005), as shown in Fig. 4.

Women have slightly higher median CPAP usage 5.2 h (IQR 0.29–8.9) compared with men 4.9 h (IQR 0.2–9.3) *p* = 0.794. Women did not have a significantly higher percentage of days with ≥ 4 h of CPAP use as described in Table 6.

**Table 6:** Gender differences in socioeconomic status and CPAP usage

Factors (Median(min, max))	Female sex (*n* = 51)	Male sex (*n* = 81)	*P*-value
Education years	14.84 (4, 20)	13.75 (4, 18)	0.128
Mean daily CPAP use, hour/night	5.2 (0.29, 8.9)	5.3 (0.2, 9.3)	0.794
Percent days above 4-hour use	73.2 (0, 100)	68.1 (0, 100)	0.201

### Clinical and polysomnographic predictors of initial CPAP acceptance

Logistic regression analysis was conducted to identify predictors of CPAP adherence (Table 7). Univariate analysis revealed that factors positively associated with CPAP acceptance included allocation to the video education group, higher educational attainment. Conversely, body mass index (BMI) was negatively associated with CPAP acceptance. Other variables, such as underlying conditions, respiratory events, and sleep parameters, showed no significant association with initial CPAP acceptance. We observed that socioeconomic factors, such as marital status, having a bed partner, and being under the Civil Servant Medical Benefit Scheme (CSMBS) with partial CPAP reimbursement, exhibited a p-value significantly greater than 0.25 in univariate analysis. Therefore, we did not include these factors in the multivariate analysis.

**Table 7:** Factors associated with CPAP acceptance using univariate logistic regression analysis

Factors	CPAP acceptance (*n* = 78)		
	OR	95% CI	*P*-value
Video intervention	2.143	1.056–4.439	0.035
Age	1.019	0.995–1.044	0.119
Male gender	0.682	0.331–1.404	0.299
BMI < 30 kg/m^2^	2.361	1.160–4.808	0.018
CSMBS	1.458	0.726–2.929	0.289
Hypertension	1.325	0.651–2.695	0.438
Diabetes mellitus	0.662	0.299–1.464	0.623
Married	0.988	0.490–1.994	0.974
Have bed partner	1.216	0.605–2.447	0.583
Education years ≥ 16	2.747	1.18–6.397	0.019
Visit with family	1.440	0.628–3.303	0.389
PSG split night	1.438	0.715–2.891	0.309
AHI	0.994	0.984–1.004	0.248
LSpO_2_	1.031	0.992–1.071	0.118
3%ODI	0.992	0.982–1.002	0.133
Sleep efficiency	0.993	0.972–1.015	0.543
Arousal index	1.001	0.987–1.015	0.928
Use nasal mask	0.349	0.038–3.212	0.353
APAP pressure setting	0.569	0.248–1.304	0.138
P95 pressure	0.962	0.846–1.094	0.560
Residual AHI	0.991	0.900–1.090	0.848
Average leakage	0.992	0.973–1.011	0.393

Multivariate logistic regression identified three independent predictors of CPAP acceptance as shown in Table 8: being in the video education group (odds ratio [OR]: 2.136; 95% confidence interval [CI]: 1.01–4.48; *p* = 0.044), having ≥ 16 years of education (OR: 2.211; 95% CI: 1.05–4.66; *p* = 0.037), and BMI < 30 kg/m^2^ (OR: 2.085; 95% CI: 0.99–4.39; *p* = 0.053).

**Table 8:** Factors associated with CPAP acceptance in multivariate logistic regression analysis

Factors	CPAP acceptance (*n* = 78)		
	OR	95% CI	*P*-value
Video intervention	2.136	1.010–4.476	0.044
Education years ≥ 16	2.211	1.050–4.656	0.037
BMI < 30 kg/m^2^	2.085	0.991–4.385	0.053

### Post-trial follow-up study at 3 months

Our randomized controlled trial of a 3-minute educational video intervention was initially designed to assess CPAP adherence in the third week. However, following positive results, we further explored the impact of the video intervention on the CPAP compliance rate over three months through a retrospective chart review. We received IRB approval for minor protocol amendments.

Unfortunately, of the 132 subjects in our previous trial, only 79 patients returned to the CPAP clinic after three months. We analyzed the adherence rate of patients at three months and observed a trend toward a higher percentage of CPAP users in the video group (68.2% vs. 60%; *p* = 0.450) compared to the usual care group (Table 9). Interestingly, 43 out of 78 patients (55.1%) with good initial acceptance continued to be good CPAP users and returned to the clinic at three months, in contrast to the poor initial acceptance group, where the majority of patients (63%) were lost to follow-up, as shown in Table 10. We did not find a significant association between the Civil Servant Medical Benefit Scheme (CSMBS) and CPAP adherence rate during the post-trial follow-up (56.9% vs. 46.4%, *p* = 0.374).

**Table 9:** Post-trial follow-up study results at 3 months (*N* = 79)

Outcomes	Video education (*n* = 44)	Usual care (*n* = 35)	*P*-value
Percent meeting criteria for good adherence, n (%)	30 (68.2)	21 (60)	0.45
Mean daily CPAP use, hour/night	5.8 (1.2, 9.2)	5.9 (2.15, 8.1)	0.782
Percent days above 4-hour use	85 (15, 100)	78 (21, 100)	0.430

**Table 10:** Patterns of CPAP usage during post-trial follow-up study

	3 months from start of intervention		
	Adherent	Non-adherent	Loss to follow up
Good initial acceptance (*N* = 78), n (%)	43 (55.1)	16 (20.5)	19 (24.4)
Poor initial acceptance (*N* = 54)	8 (14.8)	12 (22.2)	34 (63)

## Discussion

Various strategies have been developed to enhance CPAP adherence, including educational, behavioral, technological, and supportive interventions [6]. Although the behavioral strategy of implementing motivation enhancement therapy (MET) has been proven to be most successful in improving CPAP adherence, it is not available in every center. Moreover, it typically requires a certain amount in each session. In contrast, educational interventions are less complex, low-cost, and less time-consuming [6, 12].

Poor CPAP adherence is often attributed to patients’ negative perceptions of CPAP’s benefits and efficacy, as well as a lack of confidence in its therapeutic effects [13]. Evidence suggests that systematic education improves CPAP adherence by 35 to 50 min [6]. Despite the insignificant results of the previous studies, we still decided to evaluate the impact of educational videos on CPAP usage in Thai patients, which is low cost and requires lower manpower.

Our study defined the primary outcome as CPAP adherence rates at three weeks, representing initial acceptance during the standard trial period in our clinic. We primarily aimed for a longer follow-up period; however, given the limited coverage for CPAP costs in Thailand, we decided to implement a 3-week free trial period to ensure that the healthcare coverage would not affect adherence rates. We hypothesized that initial adherence could predict long-term compliance, consistent with prior findings by Coetzer et al., who demonstrated that CPAP use at one month is an independent predictor of adherence at 12 months (effect estimate ± standard error: 0.65 ± 0.07 h increase per day, *p* < 0.001) [14]. The Thai healthcare coverage program includes the Civil Servant Medical Benefit Scheme (CSMBS), social security, and a universal coverage scheme. OSA patients with CSMBS are eligible for partial reimbursement for CPAP machines, while those without CSMBS are not [15]. Thailand is currently expanding the cost coverage of CPAP for OSA patients through another healthcare scheme.

During the post-trial follow-up period, we did not find a significant difference in CPAP usage between the two groups at three months, likely due to the high dropout rate. Despite our initial concern, there was no clear association between 3-month CPAP adherence and the healthcare coverage program. One interesting finding in our study was that more than half of the patients who initially accepted CPAP at 3 weeks were also adherent at 3 months. Conversely, most patients with poor initial CPAP trials did not return to the clinic or were non-adherent, consistent with a recent study by Dielesen et al., which demonstrated that 98% of participants who were CPAP non-adherent at 3 months were likewise non-users during the first month [16]. This suggests the need for close attention and further intervention for patients with poor initial CPAP usage patterns during the first month.

While prior studies have shown mixed results regarding the efficacy of video-based education on CPAP adherence [9, 17], our results indicate significantly improved CPAP compliance in the video education group, with higher rates of adherence, average hours of CPAP usage, and the percentage of days with ≥ 4 h of use compared to the usual care group. Our findings suggest that a concise, targeted intervention may hold greater potential.

The educational video was designed based on Albert Bandura’s Social Cognitive Theory (SCT), which emphasizes self-efficacy, risk perception, and outcome expectations. These principles were incorporated to empower patients by building confidence in managing treatment challenges and understanding the benefits of CPAP therapy [11]. Potential mechanisms through which the video intervention improved adherence include increased self-efficacy and a better understanding of CPAP benefits conveyed through content that was specially developed and organized based on SCT.

Wiese et al. found that OSA patients with educational video intervention had a higher rate of return at the clinic. However, objective CPAP adherence was not available for most of the patients [17]. It is shown that the adherence rate to CPAP therapy tends to be improved by visual education, although not significant in a study by Basoglu et al. [9].

Interestingly, we observed that the educational videos used as an intervention in those studies lasted between 10 and 15 min, whereas our video was just 3 min long [9, 17]. Short-form video content has been a mainstay in digital marketing strategies for quite some time. As highlighted in previous studies [18–20], various advantages of short-form videos include the ability to maintain higher user engagement with minimal display time. Guo et al. identified video length as the most significant indicator of engagement. Videos with a duration of no longer than three minutes achieved the highest engagement rates on the Massive Open Online Course (MOOC) platform [21]. Our proposed theory suggests that the short duration of the video in this study might explain its effectiveness in capturing the audience’s attention and yielding more satisfying results. Notably, the brevity of our three-minute video may have improved participant engagement compared to the longer videos used in other studies.

The results were not significant in a recent study that evaluated the impact of a similar randomized controlled trial using a brief video on CPAP adherence in a population of patients at increased risk for poor CPAP adherence [22]. The video by Guralnick et al. lasted 4 min and focused on understanding OSA and CPAP. One explanation for the improved outcomes in the present study compared to Guralnick et al. could be the incorporation of social cognitive theory (SCT) into a short video. According to Dong et al., the success of a short video strategy does not only rely on the brevity of the video but also on how the content engages the audience’s attention [20].

Socioeconomic factors such as civil status, educational attainment, and income have been shown to influence CPAP adherence in patients with OSA [23]. Lewis et al. discovered that having a bed partner was linked to higher CPAP adherence [24], whereas being unmarried or divorced was more likely to result in non-adherence to CPAP [23]. We did not gather income data, which could affect both educational level and CPAP adherence. However, in our sensitivity analysis regarding marital status, the educational video intervention and education level continued to be significant independent predictors of CPAP adherence. The influence of these two factors on the initial acceptance of CPAP was confirmed through a multivariate logistic regression analysis that adjusted for the same covariates.

One plausible explanation for why education level might influence adherence is that people with high literacy typically possess better self-efficacy, personal judgment, and awareness of the consequences of OSA. They are more likely to understand how CPAP can reduce their risks and enhance their quality of life. Additionally, CPAP users often require problem-solving skills to address complications associated with using CPAP, such as mask leakage or dry throat, and to interpret device information sheets [25].

The effectiveness of video education interventions may differ among populations. Studies involving patients with low literacy or limited previous exposure to sleep physicians have shown less significant improvements from educational videos [22]. However, the AHEAD trial demonstrated better CPAP adherence following audio-visual health educational materials in patients with less than a high school education [26]. Their 15-minute video was specifically designed for individuals with low literacy by providing clear and engaging messages about OSA and CPAP therapy.

Although most of our participants have graduated from college, suggesting relatively higher literacy compared to previous studies, we believe our video will offer similar benefits to the low literacy group, as it was developed using simple language and clear descriptions.

In terms of gender differences, female gender was a risk factor for non-adherence to CPAP treatment at follow-up in a previous study [27]. In our study, women did not have significantly different education or socioeconomic status from men. We also did not find that each gender was positively associated with CPAP acceptance. Thus, the brief video interventions are likely applicable to both genders.

Contrary to some previous studies [14, 27] linking higher BMI to better CPAP compliance, our analysis showed that OSA patients with lower BMI had a trend towards early adherence, a conclusion backed by recent literature [28]. Sopkova et al. found that high BMI is associated with excessive mask leakage, which can result in poor CPAP adherence [29].

First, the three-week follow-up period does not reflect long-term adherence, and further studies with extended follow-up are necessary to assess the sustained efficacy of the brief educational video intervention, particularly after CPAP reimbursement is extended to other Thai healthcare coverage programs. Second, our study is single-centered, and our participants’ relatively high education level may limit the generalizability of our findings to populations with lower literacy or different cultural backgrounds. Lastly, we did not examine self-efficacy in each participant; thus, the actual relationship between their self-efficacy and CPAP adherence could not be confirmed.

In conclusion, this study highlights the potential of a video-based educational intervention added with standard care to enhance short-term CPAP adherence among OSA patients. The brevity of the video promotes engagement, while details inspired by social cognitive theory motivate positive health beliefs. By offering a low-cost, time-efficient, and scalable solution, this approach addresses key barriers to CPAP compliance. Tailored educational interventions could further improve CPAP adherence by catering to the unique needs of different demographic subgroups. However, further research involving larger and more diverse populations, particularly those with low literacy rates and extended follow-up periods, is necessary to validate its long-term efficacy and generalizability.

## Data Availability

The data supporting the findings of this study are not openly accessible due to confidentiality concerns and can be obtained from the corresponding author upon a reasonable request.

## Abbreviations

OSA: Obstructive sleep apnea
BMI: Body Mass Index
ESS: Epworth Sleepiness Scale
FSS: Fatigue Severity Scale
PSQI: Pittsburg Sleep Quality Index
AHI: apnea hypopnea index
LSpO_2_: lowest oxygen saturation from polysomnography
ODI: 3% oxygen desaturation index
SE: Sleep Efficiency
APAP: Auto-titrating Positive Airway Pressure.
